# Deep whole-genome sequencing reveals recent selection signatures linked to evolution and disease risk of Japanese

**DOI:** 10.1038/s41467-018-03274-0

**Published:** 2018-04-24

**Authors:** Yukinori Okada, Yukihide Momozawa, Saori Sakaue, Masahiro Kanai, Kazuyoshi Ishigaki, Masato Akiyama, Toshihiro Kishikawa, Yasumichi Arai, Takashi Sasaki, Kenjiro Kosaki, Makoto Suematsu, Koichi Matsuda, Kazuhiko Yamamoto, Michiaki Kubo, Nobuyoshi Hirose, Yoichiro Kamatani

**Affiliations:** 1 Department of Statistical Genetics, Osaka University Graduate School of Medicine, Suita, 565-0871 Japan; 2 Laboratory for Statistical Analysis, RIKEN Center for Integrative Medical Sciences, Yokohama, 230-0045 Japan; 3 Laboratory of Statistical Immunology, Immunology Frontier Research Center (WPI-IFReC), Osaka University, Suita, 565-0871 Japan; 4 Laboratory for Genotyping Development, RIKEN Center for Integrative Medical Sciences, Yokohama, 230-0045 Japan; 5 Department of Allergy and Rheumatology, Graduate School of Medicine, the University of Tokyo, Tokyo, 113-8655 Japan; 6 Department of Biomedical Informatics, Harvard Medical School, Boston, MA 02115 USA; 7 Department of Otorhinolaryngology—Head and Neck Surgery, Osaka University Graduate School of Medicine, Osaka, 565-0871 Japan; 8 Center for Supercentenarian Medical Research, Keio University School of Medicine, Shinanomachi 35, Shinjuku-ku, Tokyo 160-8582 Japan; 9 Center for Medical Genetics, Keio University School of Medicine, Shinanomachi 35, Shinjuku-ku, Tokyo 160-8582 Japan; 10 Department of Biochemistry, Keio University School of Medicine, Shinanomachi 35, Shinjuku-ku, Tokyo 160-8582 Japan; 11 Department of Computational Biology and Medical Sciences, Graduate school of Frontier Sciences, The University of Tokyo, Tokyo, 108-8639 Japan; 12 Laboratory for Autoimmune Diseases, RIKEN Center for Integrative Medical Sciences, Yokohama, 230-0045 Japan; 13 RIKEN Center for Integrative Medical Sciences, Yokohama, 230-0045 Japan; 14 Center for Genomic Medicine, Kyoto University Graduate School of Medicine, Sakyo-ku, Kyoto 606-8507 Japan

## Abstract

Understanding natural selection is crucial to unveiling evolution of modern humans. Here, we report natural selection signatures in the Japanese population using 2234 high-depth whole-genome sequence (WGS) data (25.9×). Using rare singletons, we identify signals of very recent selection for the past 2000–3000 years in multiple loci (ADH cluster, MHC region, *BRAP-ALDH2*, *SERHL2*). In large-scale genome-wide association study (GWAS) dataset (*n* = 171,176), variants with selection signatures show enrichment in heterogeneity of derived allele frequency spectra among the geographic regions of Japan, highlighted by two major regional clusters (Hondo and Ryukyu). While the selection signatures do not show enrichment in archaic hominin-derived genome sequences, they overlap with the SNPs associated with the modern human traits. The strongest overlaps are observed for the alcohol or nutrition metabolism-related traits. Our study illustrates the value of high-depth WGS to understand evolution and their relationship with disease risk.

## Abstract (web summary)

Recent natural selection left signals in human genomes. Here, Okada et al. generate high-depth whole-genome sequence (WGS) data (25.9×) from 2,234 Japanese people of the BioBank Japan Project (BBJ), and identify signals of recent natural selection which overlap variants associated with human traits.

## Introduction

Elucidation of natural selection signatures provides us a key to understanding the adaptive evolution of modern human populations, as well as the genetic risk of human traits^1^. Given dense mapping of the variants obtained through high-throughput single-nucleotide polymorphism (SNP) array and whole-genome sequencing (WGS), a variety of analytical methods, such as *F*-statistics (*F*_ST_)^2^, integrated haplotype score (iHS)^3^, cross-population extended haplotype homozygosity (XP-EHH)^4^, and composite of multiple signals (CMS)^5^, have been developed to fine-map natural selection signatures embedded in the human genome sequences. These methods have successfully detected genetic loci under extensive natural selection, which highlighted relationship between human evolution and both monogenic traits (e.g., lactose tolerance at *LCT* in Europeans^6^, high-altitude adaptations at *EPAS1* in Tibetans^7^, and malaria resistance at *HBB* in Africans^8^) and polygenic traits (e.g., anthropometric traits^9–11^).

These methods explore long-range haplotypes consisting of common variants and diversity in derived allele frequency (DAF) spectra; therefore, positive selection pressure which corresponds to relatively older ages of modern human history from 250,000 to 30,000 years ago has been mostly examined^1^. However, worldwide human populations have separately experienced expansions of their effective sizes under different environments during more recent ages such as the last 10,000 to 20,000 years^12^, and such adaptations may be more closely related to epidemiology of human diseases today. Thus, a comprehensive assessment of very recent selection pressures is warranted. Further, since some of these methods interrogate multiple populations to enhance their power to detect sweeps^2,4,5^, the interpretation of the results will be dependent on the set of examined populations, which complicates the assessment of selection pressures in a single population of interest.

Recently, Field et al.^9^ developed a novel method named singleton density score (SDS), which can detect the signatures of very recent natural selection in a single population using WGS data. SDS handles intervals between each common variant and the nearest singletons detected by WGS as measures to distinguish selection from neutral drift. Singletons generally appeared more recently than common variants in the population, which allows SDS to infer natural selection from more recent time periods than the previous methods that handle common haplotypes. While the timeframe depends on the sample size and demographic history, an estimated resolution of SDS is approximately considered to be 100 generations (i.e., around the past 2,000–3,000 years). Application of SDS to the WGS data of 3195 individuals in the UK10K project^13^ identified strong selection signatures in multiple loci, including *LCT* and the major histocompatibility complex (MHC) region with implication in human complex traits such as pigmentation and adult height, respectively. While credible assessment of singletons requires costly high-depth WGS data, additional application of SDS to non-European populations would contribute to our understanding of evolutional history of humans.

In this study, we report natural selection signatures in the Japanese population using high-depth WGS data of the Japanese ancestry (*n* = 2234). We apply the SDS method to identify genetic loci under very recent natural selection signatures with corroborative assessment of the population structure of Japanese using large-scale genome-wide association study (GWAS) data and principal component analysis (PCA; *n* = 171,176). Moreover, we examine selection signature profiles on human genome sequences derived from archaic hominins, as well as risk variants on a range of modern human complex traits, to assess underlying impacts of adaptive evolution in Japanese.

## Results

### High-depth WGS of 2234 Japanese individuals

**Fig. 1:**
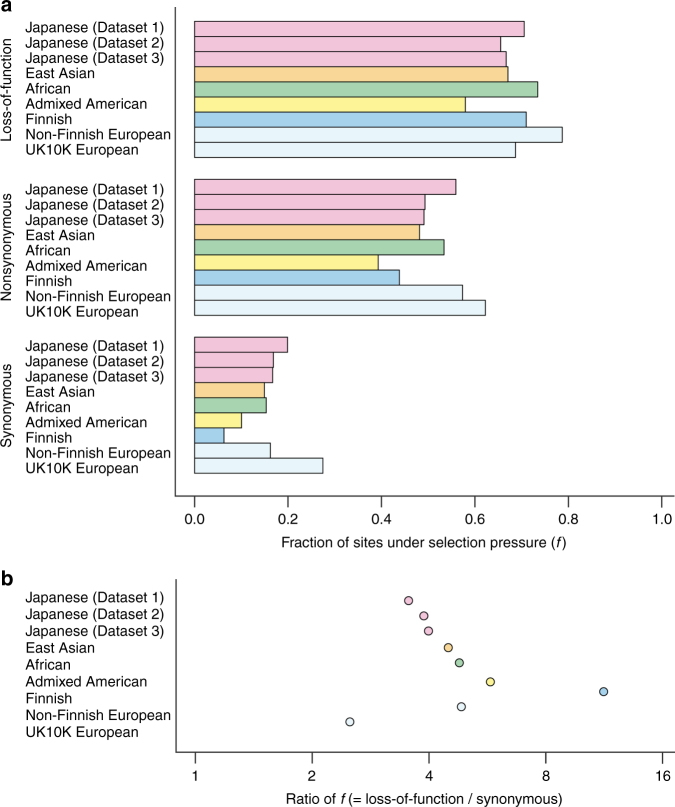
Site frequency spectrum and fraction of sites under selection in the worldwide populations. Site frequency spectrum (SFS) estimated for the worldwide populations. In addition to the Japanese WGS datasets, data obtained from the Genome Aggregation Database (gnomAD; African, admixed American, east Asian, Finnish, and Non-Finnish European) and the UK10K project (European) are indicated^13,17^. **a** Fraction of sites under selection pressure ( = *f*) calculated separately for loss-of-of-function variants, nonsynonymous SNV, or synonymous SNV. **b** Ratio of *f* between loss-of-of-function variants and synonymous SNV

**Fig. 2:**
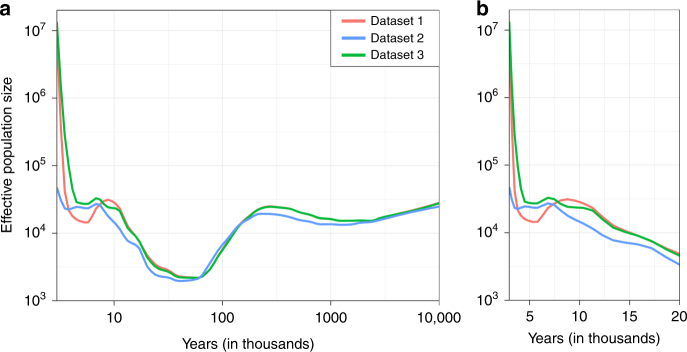
Longitudinal change of the effective population size of the Japanese population. Longitudinal change of the effective population size of the Japanese population estimated from the WGS data. The effective population sizes were estimated separately for the datasets 1–3, using SMC++ software^20^. Times are indicated in **a** logarithm and in **b** linear scales. One generation was considered to be 29 years

We conducted WGS in a total of 2234 individuals of Japanese ancestry, most of whom were enrolled from the BioBank Japan Project (BBJ; Supplementary Table 1)^14,15^, which recruited patients from the nation-wide regional populations of Japan^16^. We integrated in total three WGS datasets (*n* = 1276, 492, and 466, respectively), all of which achieved high-sequence depth of the called variants (weighted mean 25.9×). After applying the stringent variant filtering procedure, we obtained a total of 39,898,568 autosomal variants (Ti/Tv ratio = 2.11–2.14). Minor allele frequency (MAF) spectra indicated that 74.7% of the variants were rare (MAF < 0.5% in any of the datasets). Proportions of the loss-of-function variants were higher for the variants with lower alternative allele frequencies (Supplementary Fig. 1), which suggests selection pressure on these variants. Thus, we assessed site frequency spectrum (SFS) of the WGS datasets, as well as those in worldwide populations obtained from the Genome Aggregation Database (gnomAD)^17^ and the UK10K project^13^ (Fig. 1a and Supplementary Fig. 2). As expected from previous findings^18^, a ratio of the fractions of sites under selection pressure (= *f*)^19^ between loss-of-function variants and synonymous SNV was high in the Finnish population ( = 11.3), while the Japanese population indicated moderate values ( = 3.5–4.0; Fig. 1b). Effective population sizes estimated from the WGS datasets showed rapid increases over approximately the past 10,000 years, as also reported in other populations (Fig. 2)^20^. To our knowledge, this is one of the largest high-depth WGS data studies ever reported in a single non-European population.

### Genome-wide natural selection signatures in Japanese

Using the high-depth WGS data, we evaluated genome-wide natural selection signatures of the Japanese population. We calculated SDS to evaluate very recent selection signatures (around the past 2000–3000 years). Since SDS calculation requires accurate information on genome-wide localization of the singletons, we excluded the genomic regions with low confidence in variant calling from the subsequent analysis (e.g., centromeres or regions with an excess density of singletons).

**Fig. 3:**
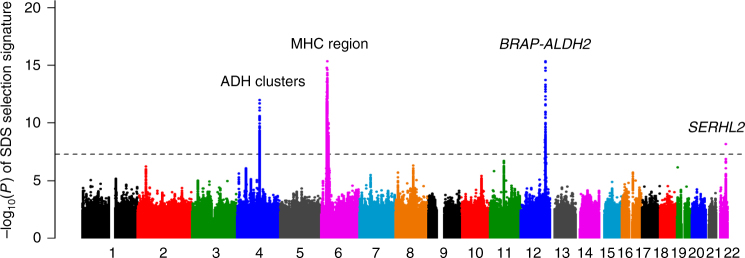
Genome-wide very recent natural selection signatures of the Japanese population. A Manhattan plot of the genome-wide natural selection signatures obtained from the WGS data of 2234 Japanese individuals. The *y*-axis indicates the –log_10_(*P*) of a genome-wide selection signature calculated by using SDS^9^. The horizontal gray line represents the genome-wide significance threshold (*P* < 5.0 × 10^-8^)

By meta-analyzing the results of the three WGS datasets, we obtained normalized SDS (and corresponding *P*-values) for 6,292,092 common variants, respectively, (MAF ≥ 0.01; Fig. 3 and Supplementary Fig. 3). The genome-wide SDS *P*-values demonstrated significant natural selection pressure that satisfied the genome-wide significance threshold (*P* < 5.0 × 10^−8^)^21^; alcohol dehydrogenase (ADH) gene clusters at 4q23 (rs75721934, *P* = 9.7 × 10^−13^), MHC region at 6p21 (rs58008302, *P* = 4.1 × 10^−16^), *BRAP-ALDH2* at 12q24 (rs3782886, *P* = 4.4 × 10^−16^), and *SERHL2* at 22q13 (rs4822159, *P* = 6.6 × 10^−9^; Table 1 and Supplementary Fig. 4). The ADH cluster, MHC region, and *BRAP-ALDH2* showed strong selection signatures (*P* < 1.0 × 10^−12^) that spanned long distances within the region (0.24 Mbp, 3.57 Mbp, and 2.44 Mbp for the SNPs with genome-wide significance), which was highlighted as a long LD block within the loci (Supplementary Fig. 4). These results empirically suggest that natural selection signatures in the Japanese population were most evident at these three loci, which were distinct from observations in Europeans^9^.

**Table 1:** SNPs with very recent natural selection signatures in the Japanese population

rsID	Chr	Position (hg19)	Ancestral derived	DAF in WGS	Gene	Selection signature		DAF heterogeneity			
								1000 Genomes Project global (*n = *2504)		Japanese (*n* = 171,176)	
						*z*-score	*P*	Fold change	*P*	Fold change	*P*
SNPs with SDS selection signatures (2234 Japanese subjects)											
rs75721934	4	100,142,780	G/A	0.750	ADH clusters	7.13	9.7 × 10^−13^	10.26	8.5 × 10^−6^	4.32	0.021
rs58008302	6	29,493,261	G/A	0.186	MHC region	8.14	4.1 × 10^−16^	0.61	0.63	14.82	7.4 × 10^−5^
rs3782886	12	112,110,489	T/C	0.289	*BRAP*-*ALDH2*	8.13	4.4 × 10^−16^	3.66	0.0044	12.17	1.5 × 10^−5^
rs4822159	22	42,932,013	C/G	0.193	*SERHL2*	−5.80	6.6 × 10^−9^	0.99	0.40	3.41	0.045

DAF, derived allele frequency; WGS, whole-genome sequence

The top SNPs from the SDS analysis in ADH cluster and *BRAP-ALDH2* are in linkage disequilibrium (LD) with functional missense SNPs of *ADH1B* (Arg47His, rs1229984) and *ALDH2* (Glu504Lys, rs671) (*r*^*2*^ = 0.76 and 0.86 in the WGS dataset 1, respectively). These SNPs mediate differences in alcohol metabolism by altering the enzymatic activities of alcohol dehydrogenase and aldehyde dehydrogenase, and have been suggested in evolutional adaptations of global populations including Asians mostly by candidate gene-based approaches^22^. Our genome-wide analysis initially indicates that both of these alcohol metabolism-related SNPs are under the strongest recent natural selection pressure in the Japanese population. The MHC region includes multiple human leukocyte antigen (HLA) genes, which mediate immune responses, and the top SNP of the SDS analysis was located within the extended MHC class I region^23^. The alleles and haplotypes of the HLA genes are known to be under population-specific selection pressure, which was highlighted as frequency spectra heterogeneous among populations^24–28^. *SERHL2* belongs to the serine hydrolase family, while its functional role is yet to be elucidated^29^, and other nearby genes in the region, such as *RRP7A*, *RRP7B*, and *POLDIP3*, could also be biological candidates linked to selection pressure.

### DAF heterogeneity of the SNPs with selection signatures

Since natural selection signatures induce rapid allele frequency changes^30^, we assessed whether the top SNPs detected by the genome-wide SDS analysis were enriched for DAF spectra heterogeneity in the Japanese population. We quantitatively examined DAF heterogeneity enrichment using large-scale BBJ GWAS data of Japanese ancestry consisting of seven regional residents of Japan (*n* = 171,176)^31,32^. These geographic regions are located from the northeast to southwest parts of Japan (Hokkaido, Tohoku, Kanto-Koshinetsu, Chubu-Hokuriku, Kinki, Kyushu, and Okinawa), as described elsewhere^16^. We also evaluated five subpopulations in the global subjects of the 1000 Genomes Project Phase 3 data (Africans [AFR], admixed Americans [AMR], East Asians [EAS], Europeans [EUR], and South Asians [SAS], *n* = 2504)^33^.

**Fig. 4:**
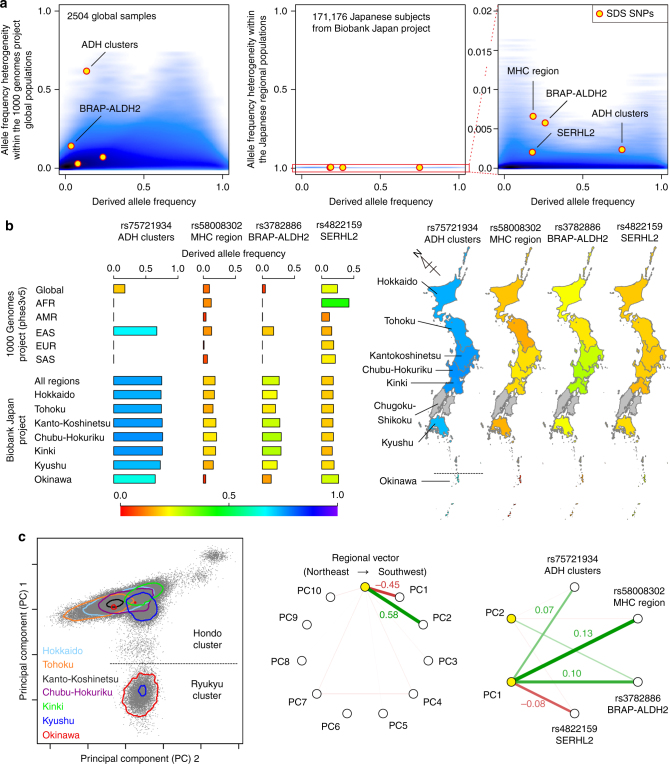
Derived allele frequency heterogeneity of the SNPs with natural selection signatures. **a** DAF heterogeneity of the SNPs within subpopulations of the 1000 Genomes Project global subjects, or the regional populations of the Japanese subjects from the BBJ cohort. Strength of blue color corresponds to the density of the SNPs. Circles indicate the top SNPs identified by SDS, and the top SNPs with nominally significant enrichment of DAF heterogeneity are labeled (*P* < 0.05). **b** DAF spectra of the four SNPs with genome-wide SDS selection signatures in each sub- or regional populations. DAF in each of the seven regions of Japan (Hokkaido, Tohoku, Kanto-Koshinetsu, Chubu-Hokuriku, Kinki, Kyushu, and Okinawa) are colored in the geographical map. We note that DAF in Chugoku-Shikoku was not available (colored in gray). **c** Correlations among the regional vector of Japan, PCs, and the SDS top SNP genotypes. PC1 separated the Japanese population into the two major clusters, Hondo and Ryukyu (left panel). Correlations between the regional vector and each of PCs (middle panel), and between top two PCs and each of the top SNP genotypes from the SDS analysis (right panel) are indicated. PC1 showed strong correlations with the regional vector and the SNP genotypes

Regarding the 1000 Genomes Project global subjects, the SDS-identified top SNPs in ADH clusters and *BRAP-ALDH2* showed nominally significant excess of DAF heterogeneity (*P* < 0.0044 for DAF heterogeneity enrichment test; Table 1 and Fig. 4a). Particularly, the SNP within ADH cluster (rs75721934) showed as high as 10.26-fold of heterogeneity enrichment after adjustment by the corresponding DAF bin, being the highest DAF = 0.672 in EAS but having negligible frequencies in other populations (DAF ≤ 0.001; Fig. 4b and Supplementary Table 2). Among the five EAS subpopulations, the highest DAF was observed in the Japanese population (JPT, DAF = 0.731). Relatively high DAF were also observed in the Chinese (DAF = 0.601, 0.670, and 0.714 for CDX, CHB, and CHS, respectively) and Vietnam (KHV, DAF = 0.626) populations (Supplementary Table 2). This supports the previous findings that the functional variant of ADH cluster region is under selection in the east Asian populations^3,34^. Regarding the Japanese BBJ subjects, all the four SDS top SNPs within ADH cluster, MHC region, *BRAP-ALDH2*, and *SERHL2* showed significant DAF heterogeneity enrichment among the Japanese geographic regions (*P* < 0.05 for DAF heterogeneity enrichment test). These results empirically suggested that very recent selection pressure captured by the SDS analysis directly reflects recent DAF changes of genetic landscape in the Japanese population.

DAF heterogeneity among the SDS-identified top SNPs was most evident in Okinawa, the most southwest islands of Japan, with the highest DAF at *SERHL2* and the lowest DAF for the other three loci (Fig. 4b). Since the genetic architecture of the population represented by PCA is related to its geographic localization^35^, we assessed correlations between the regional vector of Japan (from northeast to southwest) and each of the top ten principal components (PCs). As reported previously, PC1 and PC2 correlated with the regional vector, and PC1 separated the Japanese population into two major clusters, Hondo (the main islands of Japan) and Ryukyu (the Okinawa islands; Fig. 4c and Supplementary Fig. 5)^16,36^. *F*_ST_ between these two clusters was 0.0047. All the SDS-identified top SNPs showed strong correlations with PC1, suggesting that heterogeneous geographic adaptations between these two clusters could partially explain DAF heterogeneity induced by very recent selection pressures on the Japanese population.

### Selection signature in archaic hominin-derived sequences

Inheritance of genomes from archaic hominins to modern humans, such as from Neanderthals and Denisovans, and its impact on the adaptation of human complex traits, is of major importance of human genetics and anthropology^37–40^. We, thus, evaluated natural selection signature profiles within three sets of Neanderthal-derived sequences reported in the Japanese population^37–39^ using the permutation analysis in which null distribution of the test statistics was empirically estimated from those calculated by the genome-wide sliding window approach. We did not observe significant shift of the mean SDS *z*-scores (*P* > 0.30; Supplementary Fig. 6), which might suggest that Neanderthal-derived sequences were not apparently under significant selection pressure in the recent ages in the Japanese population.

### Selection signature at Japanese GWAS-associated variants

Adaptations of the human populations are closely related to the risk of phenotypes that affect survival. Therefore, we evaluated enrichment of the SDS selection signatures on the variants associated with human complex traits in Japanese. We collected the 1594 Japanese GWAS-associated variants of 97 traits which consist of diseases (*n* = 36) and quantitative traits classified as 12 categories (anthropometric [*n* = 2], behavior [*n* = 2], blood pressure [*n* = 4], echocardiographic [*n* = 5], electrolyte [*n* = 5], hematological [*n* = 13], kidney-related [*n* = 4], liver-related [*n* = 6], metabolic [*n* = 6], other biochemical [*n* = 8], pharmacogenetics [*n* = 2], and protein [*n* = 4])^31,32,41–44^. The 9712 variants with immune-cell-specific expression quantitative trait loci (eQTL) effects identified in the Japanese population were also collected (CD4^+^ T cells, CD8^+^ T cells, B cells, natural killer cells, monocytes, and unfractionated peripheral blood; *n* = 6)^45^. To comprehensively conduct the phenome-wide screening, we evaluated selection signature enrichment regardless of consistency of allelic directional effects on the traits.

**Fig. 5:**
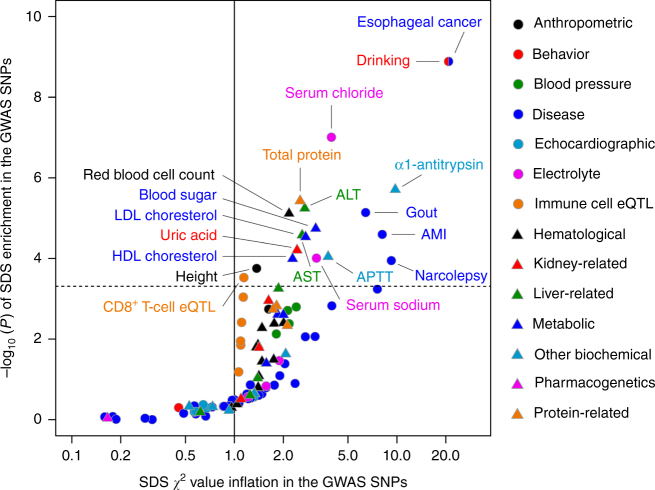
Overlap between natural selection signatures and genetic risk of human phenotypes in Japanese. Enrichment of the natural selection signatures in the GWAS-associated variants of the diseases (*n* = 36), quantitative traits (*n* = 61), and immune-cell-specific eQTL (*n* = 6) in Japanese. For each trait, inflation of the selection *χ*^2^ value is indicated along the *x*-axis, and –log_10_(*P*) of enrichment is plotted along the *y*-axis. The horizontal gray line represents significance threshold based of Bonferroni’s correction on the numbers of the evaluated traits (*P* < 0.00049)

Of these, 19 traits showed significant enrichment of overlap between the trait-associated SNPs and the SDS selection signatures (*P* < 0.05/(97 + 6) = 0.00049 for selection overlap enrichment; Fig. 5 and Supplementary Table 3). As expected, phenotypes that have relationships with the variants within the three major selection signature loci (ADH cluster, MHC region, and *BRAP-ALDH2*) showed significant enrichment. The most significant phenotypic enrichment was observed for drinking behavior (i.e., alcohol consumption dose) and esophageal cancer (*P* = 1.3 × 10^−9^), the two traits with strong genetic risks linked to the functional missense SNPs at the alcohol metabolism-related genes (*ADH1B* and *ALDH2*)^41,42^. Missense alleles of *ADH1B* (Arg47His) and *ALDH2* (Glu504Lys) associated with lower alcohol consumption doses both indicated strong positive selection pressure in Japanese^41,42^. While, it would be difficult to examine whether drinking itself was a causal phenotype that derived selection pressure. Considering that drinking is a major risk factor of esophageal cancer, regional distributions of DAF spectra of the *ADH1B* and *ALDH2* functional alleles partly explain its similarity with those of drinking habit, and as a consequence, prevalence of esophageal cancer^22,41^. Other diseases such as gout^43^ also showed significant overlap with the selection signatures. Previous studies reported contribution of multiple population-specific rare variants on gout and hyperuricemia susceptibility in Japanese^46^. Our findings would suggest that accumulation of the rare variants associated with these traits has been accelerated by regional natural selection pressures.

Regarding the quantitative traits, previous European studies suggested enrichment of the natural selection signatures in the variants associated with the anthropometric (height and obesity)^9–11^ or immune-related traits (immune responses to pathogens)^47,48^. We replicated natural selection profiles on some of these traits in the Japanese population (*P* = 0.00017 for height and *P* = 0.00030 for CD8^+^ T cell-specific eQTL). However, quantitative traits related to nutrition metabolisms showed more evident overlap with selection signatures than anthropometric or immune-related traits (i.e., total protein, blood sugar, high-density lipoprotein [HDL] and low-density lipoprotein [LDL] and cholesterols, electrolytes, liver enzymes, and uric acid; *P* ≤ 1.0 × 10^−5^), which was distinct from previous findings in Europeans. We then evaluated selection enrichment overlap when four genomic loci with genome-wide significant selection signature were removed. While magnitudes of enrichment reduced, several traits related to nutrition metabolisms still showed overlaps (e.g., protein-related traits, lipids, electrolyte, and gout, FDR-*q* < 0.05; Supplementary Fig. 7).

Selection signatures of the traits reflect regional adaptation in each population (e.g., northern adaptation of height for high latitude within the European continent^10^). Thus, our findings suggest that: (i) selection pressures on human complex traits could be differently characterized between Europeans and Japanese; and (ii) nutrition metabolisms as well as alcohol metabolisms have played important roles in recent adaptation of the Japanese population.

## Discussion

In this study, we evaluated natural selection pressure on the Japanese population during very recent ages (the past 2000–3000 years), using high-depth large-scale WGS data (25.9×) of over 2200 individuals. Subsequent analysis integrating the GWAS data of over 170,000 subjects demonstrated a close relationship of the identified selection signatures with the Hondo and Ryukyu regional clusters of the Japanese population. While the recent selection signatures in the Japanese population did not show apparent enrichment in Neanderthal-derived sequences, clear overlaps with the genetic risk of the human phenotypes, especially those of the alcohol- or nutrition metabolism-related traits, were observed.

Our study reports several novel findings. First, this is the largest high-depth WGS data study ever conducted on a single, non-European population. Previous studies have reported the benefit of high-depth WGS for rare variant detection and improved imputation accuracy^49,50^. Moreover, our study demonstrated its advantage in the studies of human evolution by utilizing the singleton variants. Secondly, we identified multiple loci with strong, very recent selection signatures in Japanese (ADH cluster, MHC region, and *BRAP-ALDH2*). These loci were different from previous findings in Europeans, which indicates the necessity of investigating additional populations by WGS to examine human evolution. While our WGS data includes the disease patients of the BBJ cohort, we note that the selection signals of these loci were still significant even when conditioned on the disease affection status, suggesting that disease affection status itself may not have biased the results. Thirdly, the identified very recent selection signatures were independently validated by utilizing DAF spectra heterogeneity and PCA of the large-scale GWAS data. While the current next-generation sequencing (NGS) technology still has limitations in its quality, this consistency between different approaches greatly reduces the possibility that the observed selection signatures resulted from bias introduced by variant calling errors of the WGS data. Fourthly, contrary to our expectations, very recent selection signatures in the Japanese population were not enriched in Neanderthal-derived sequences. Our results raise further questions on interplay between archaic hominins and modern humans. Finally, we found overlaps between very recent selection signatures and human phenotype genetic risk in Japanese, specifically for alcohol or nutrition metabolism-related traits that were clearly distinct from those found in Europeans and Africans highlighted as anthropometric or immune response-related traits. This provides novel insights into the process of modern human evolution with regard to evolutional circumstances specific to each population.

Previous genetic studies have assessed geographical adaptation of the Japanese population mostly from the following two aspects: on distinct clusters within modern Japanese geographical localizations (Hondo and Ryukyu)^16^; and on admixture history of ancient Japanese lineages (e.g., Jomon and Yayoi)^51^. Our study provides empirical evidence on the former aspect in relation to very recent natural selection pressures in Japan, while further accumulation of ancient Japanese genome sequences will be necessary to unbiasedly assess the latter aspect. We also note that our PCA analysis could be overestimating the differences observed for distinct Hondo and Ryukyu clusters, due to the relatively higher proportion of the Okinawa residents in the BBJ cohort (3.3%), as compared with the actual proportion in the Japanese population (1.1%). We note that when confining the WGS samples into those belonging to the Hondo cluster (*n* = 2190), very recent selection signatures observed at all the four loci were still genome-wide significant, thereby suggesting that these selection signatures were not biasedly induced by the population structure.

In conclusion, our WGS-based analysis identified very recent selection signatures and their relationships with evolution, introgression with ancient hominins, and risk of human phenotypes in Japanese individuals. Our study highlights the value of high-depth WGS to understand human adaptations and history.

## Methods

### Subjects in the WGS analysis

We enrolled a total of 2234 individuals of Japanese ancestry for the WGS analysis. Of these, 1939 individuals were BBJ participants^14,15^ who were affected with any of the seven diseases (acute myocardial infarction, drug eruption, colorectal cancer, breast cancer, prostate cancer, gastric cancer, and dementia). The remaining 295 individuals were Japanese who lived over 100 years (Supplementary Table 1). Subjects who were determined to be of non-Japanese origin either by self-reporting or by PCA of the WGS data or of our previous study^31,32^, were excluded. All the subjects provided written informed consent as approved by the ethical committee of RIKEN Yokohama Institute and the Institute of Medical Science, the University of Tokyo (for the BBJ subjects), and Keio University (for the controls).

### High-depth WGS data analysis

WGS were conducted separately for three datasets (*n* = 1276, 492, and 466, respectively; Supplementary Table 1) with the design to achieve high-depth of the mapped reads (20–35×). DNA samples were collected and de-identified at BBJ^14,15^ and Keio University. WGS were conducted at RIKEN Center for Integrative Medical Sciences (dataset 1), Toshiba Corporation (for the BBJ subjects in the dataset 2), Takara Bio Inc. (for the controls in the dataset 2), and Macrogen Japan Corporation (dataset 3). DNA quantity was measured by Picogreen and degradation of DNA was assessed by gel electrophoresis. We selected DNA of good quality and concentration for making the DNA libraries. WGS library was constructed using the TruSeq Nano DNA Library Preparation Kit (Illumina) for the dataset 1 and the TruSeq DNA PCR-Free Library Preparation Kit for the dataset 2 and 3 according to the manufacturer’s protocols. After quantification of DNA libraries by quantitative PCR and a Bioanalyzer (Agilent Technologies), we sequenced using 2 × 160-bp paired end reads on a HiSeq2500 platform (Illumina) with rapid run mode and 2 × 125-bp paired end reads on HiSeq2500 with high output run mode for the dataset 1 (*n* = 1026 and 250, respectively), 2 × 125-bp paired end reads on HiSeq2500 with high output run mode for the dataset 2 (for the controls), and 2 × 150-bp paired end reads on a HiSeq X Five (Illumina) for the dataset 2 (for the BBJ subjects) and dataset 3.

### Variant calling of the WGS data

Variant calling of the WGS data was conducted separately for each dataset according to the following analytical pipelines. The sequence reads were converted to the FASTQ format using bcl2fastq (version 1.8.4 for the dataset 1) or bcl2fastq2 (version 2.17.1.14 for the datasets 2 and 3) and trimmed to clip Illumina adapters using Trimmomatic (version 0.36 for the datasets 2 and 3). They were aligned to the reference human genome with the decoy sequence (GRCh37/hg19, hs37d5) using BWA-MEM (version 0.7.5a). The duplicated reads were removed using picard (versions 1.106, 1.106, and 2.5.0 for the datasets 1, 2, and 3, respectively). Indel realignment and base quality score recalibration were done by using GATK (versions 3.2–2, 3.5–0, and 3.6 for the datasets 1, 2, and 3, respectively). Individual variant call results were generated using GATK HaplotypeCaller. Multi-sample joint-calling of the variants was also performed using GATK, then we filtered out genotypes, which satisfied following criteria: (1) DP < 5, (2) GQ < 20, or (3) DP > 60, and GQ < 95. We used this joint call dataset to evaluate call rate of each variant and subject, and removed variants with low genotype call rates (<0.90) and subjects with low genotype call rates (<0.99). Thereafter variant quality score recalibration was applied according to the GATK Best Practice recommendations^52^. We removed the variants located in the low complexity regions, and genotype refinement was performed using Beagle (versions 3.3.2 for the dataset 1, and version 4.1 for the datasets 2 and 3). We excluded the subjects with excess genotype heterozygosity, or excess numbers of singletons. Finally, we applied the strict mask to obtain a set of variants with high accuracy. Namely, we excluded the variants with read depths more than double or less than half of the genome-wide average depth, QUAL < 56. Variants deviating strongly from Hardy Weinberg Equilibrium (*P* < 1.0 × 10^−6^ calculated using vcftools [version 0.1.12b]) were also excluded.

We empirically confirmed accuracy of genotype calling in our WGS datasets. First, all the WGS datasets achieved high concordance rates of the genotypes with those genotyped by SNP microarrays^31,32^ (≥99.97%), which provides confidence in calling of common variants. Further, we randomly selected 1657 singletons from the WGS dataset 1, and validated them using target deep sequencing (mean depth of the target singleton sites = 705.2×), as described elsewhere^53^. We observed low false discovery rate (FDR) of 1.03%, which provides confidence in calling of rare variants. While the variant calling pipeline of each WGS dataset consists of slightly different versions of the software, we thus confirmed that differences in the pipelines did not affect the accuracy of the WGS variant calling. We note that joint-calling of all the three WGS dataset, as well as other WGS datasets in Japanese, could improve accuracy of variant calling and increase statistical power to assess selection pressure.

### Annotation of the WGS data

Functional annotations of the variants called in the WGS data were performed using ANNOVAR (version 2015Dec14) and RefSeq. We grouped together insertion/deletion, stopgain/loss, and splicing variants as loss-of-function variants to compare alternative allele frequency spectra across different functional categories. Annotation of ancestral and derived alleles was conducted according to the 1000 Genomes Project Phase3 v5 data^33^ and the dbSNP database version 150. SFS and the fraction of sites under selection pressure was calculated using original scripts of Moon et al. (see URLs)^19^. Intron and intergenic sites were used as a reference. Variant frequency data of the worldwide populations were obtained from gnomAD (*n* = 4368 for African, *n* = 419 for admixed American, *n* = 811 for east Asian, *n* = 1747 for Finnish, and *n* = 7509 for Non-Finnish European) and the UK10K project (*n* = 7652 for European)^13,17^. Effective population sizes of the subjects were estimated for each dataset separately, using WGS data of randomly selected subjects (*n* = 100 for each dataset) and SMC++ (version 1.8.0)^20^. We employed the fixed per-generation mutation rate at 1.25 × 10^−8^ mutations per base pair and a constant generation time of 29 years.

### Calculation of natural selection signatures

Using the WGS data, we calculated genome-wide natural selection signatures of the Japanese population. We identified singletons (and private doubletons) from each WGS dataset and applied filters to extract those used for inferring SDS. First, we removed the singletons located in the genomic regions where accurate variants calling using the NGS technique were empirically known to be difficult (i.e., centromeres, heterochromatins, or acrocentric chromosomes). Second, we evaluated genome-wide density of the singletons with half-overlapping sliding windows (20 kbp lengths), and removed the singletons located in the high-density windows that lay outside of +4 standard deviations.

We then calculated the SDS^9^ using original authors’ scripts (see URLs). We first estimated the gamma-shape parameters of each DAF bin (bin widths = 0.005 for DAF < 0.1 or DAF > 0.9, and bin widths = 0.01 for 0.1 ≤ DAF ≤ 0.9), based on the demographic model of “Gravel_CHB” and the number of the effective population size of 100,000. An initial guess for the maximum likelihood optimization was set at 1.0 × 10^−6^. The SDS *z*-scores of the genome-wide common variants with MAF ≥ 0.01 with available annotations of ancestral and derived alleles were calculated using the filtered singletons. The SDS *z*-scores were calculated separately for each chromosome arm and each WGS dataset. For each WGS dataset, standardized SDS *z*-scores were obtained through normalization of the genome-wide raw SDS scores within each DAF of the bins. We selected the common variants of which SDS *z*-scores were calculated for all three WGS datasets, and conducted meta-analysis of the standardized SDS *z*-scores of the WGS datasets using the *z*-score method weighted according to the square root of the number of the samples in each WGS dataset^54^. After the meta-analysis, the genome-wide SDS scores were normalized again to calculate two-tailed *P*-values for significance in natural selection signatures. We set the typical genome-wide significance threshold as a significance threshold of our study (*P* < 5.0 × 10^−8^)^21^.

### DAF heterogeneity enrichment analysis

DAF heterogeneity of the genome-wide common variants with SDS *z*-scores were calculated for the global subjects obtained from the 1000 Genomes Project Phase3 v5 data (*n* = 2504)^33^ and for the Japanese individuals acquired from the genome-wide imputed GWAS data of BBJ (*n* = 171,176)^31,32^. For each variant, DAF heterogeneity was calculated as *χ*^2^ values from the allele count contingency table that consisted of derived and ancestral alleles (rows) and subpopulations (columns), which were then divided by the total number of the alleles. This index ranges from 0 to 1, and a higher value indicates higher DAF heterogeneity. Enrichment of DAF heterogeneity for the top SNPs with genome-wide significant SDS *z*-scores was assessed using the one-tailed test, with adjustment on the DAF bins. Distribution of the heterogeneity indices obtained from the SNPs included in each DAF bin ( ± 0.005) was defined as a null distribution. Fold change of the enrichment was obtained by dividing the observed DAF heterogeneity by the mean value of the null distribution of the corresponding DAF bin.

Subpopulations of the 1000 Genomes Project data were classified as AFR (*n* = 661), AMR (*n* = 347), EAS (*n* = 504), EUR (*n* = 503), and SAS (*n* = 489) according to the definitions of the 1000 Genomes Project. Subpopulations of the BBJ subjects were defined according to recruitment sites grouped into the seven geographic regions that line the northeast to southwest parts of Japan: Hokkaido (*n* = 7910), Tohoku (*n* = 10,907), Kanto-Koshinetsu (*n* = 95,272), Chubu-Hokuriku (*n* = 9390), Kinki (*n* = 26,160), Kyushu (*n* = 15,818), and Okinawa (*n* = 5719)^16^. We note that there were no subjects recruited from the geographic region, Chugoku-Shikoku, which is located between Kinki and Kyushu. PCA data of the BBJ GWAS data were obtained from the previous study^31,32^. *F*_ST_ between the Hondo and Ryukyu clusters was calculated using smartpca (version 6.0.1), as described elsewhere^55^.

### Selection signature in archaic hominin-derived sequences

We evaluated whether human genome sequences of the Japanese population derived from archaic hominins, such as Neanderthal or Denisovan, were under natural selection pressure. We obtained genomic regions from three previous studies^37–39^, where introgressions of the sequences derived from Neanderthal were observed in the Japanese individuals (3195, 3961, and 4223 loci spanning approximately 563, 296, and 578 total Mbp, respectively). For each introgression region, we calculated mean values of the SDS *z*-scores for the variants located within the Neanderthal-derived sequences. To generate a null distribution of the mean *z*-scores, we physically slid the introgression loci along with the concatenated chromosomes, in increments of 0.01% of the total length of concatenated chromosomes (×10,000 iterations), thereby maintaining the relations among neighboring variants while shuffling the introgression flags in the Neanderthal-derived sequences. In each permutation step, mean *z*-scores of the variants within the slid introgression loci were calculated to simulate the null distribution, which was used to calculate two-sided permutation *P*-values. We note that no Denisovan-derived sequences were observed in Japanese^39^, and thus, enrichment analysis was not conducted.

### Selection signatures enrichment in the GWAS-associated SNPs

We evaluated enrichment of the natural selection signatures on the human complex trait-associated variants in the Japanese population. We collected a list of the variants identified by the GWAS conducted for the Japanese population that satisfied the typical genome-wide significance threshold (*P* < 5.0 × 10^−8^). We curated the GWAS catalog database to obtain the variant list, in addition to manual curation of the published literature^41–44^. We also integrated the GWAS results of BBJ (anthropometric traits^31^ and biochemical and hematological biomarkers)^32^ and the immune-cell-specific eQTL results of Japanese ancestry^45^. To robustly estimate enrichment, we applied rank-based normalization to the SDS *z*-scores. For each set of the GWAS-associated variants of a trait, the sum of the square values of the rank-based normalized SDS *z*-scores of the variants (or the proxy variants in LD [*r*^*2*^ > 0.5 in 1000 Genomes Project EAS]) were compared to the χ^2^ distribution with the degree of freedom equal to the number of the variants.

### Data source

The URLs for data presented herein are as follows:

The Genome Aggregation Database (gnomAD), http://gnomad.broadinstitute.org/

The UK10K project, https://www.uk10k.org/data.html

The BioBank Japan Project (BBJ), https://biobankjp.org/english/index.html

Bcl2fastq and Bsl2fastq2, https://support.illumina.com/sequencing/sequencing_software/bcl2fastq-conversion-software.html

BWA-MEM, http://bio-bwa.sourceforge.net/

Picard, https://broadinstitute.github.io/picard/

GATK, https://software.broadinstitute.org/gatk/

Beagle, https://faculty.washington.edu/browning/beagle/beagle.html

Vcftools, http://vcftools.sourceforge.net/

ANNOVAR, http://annovar.openbioinformatics.org/en/latest/

Fraction under selection, https://github.com/moon-s/fraction-under-selection

The 1000 Genomes Project, http://www.internationalgenome.org/

dbSNP, https://www.ncbi.nlm.nih.gov/projects/SNP/

SMC++, https://github.com/popgenmethods/smcpp

Singleton density score (SDS), https://github.com/yairf/SDS

GWAS catalog, https://www.ebi.ac.uk/gwas/

Immune-cell-specific eQTL results, https://humandbs.biosciencedbc.jp/hum0099-v1

JENGER, http://jenger.riken.jp

### Data availability

WGS data of a part of the BBJ subjects (*n* = 1,026) is publically available at the National Bioscience Database Center (NBDC) Human Database (https://humandbs.biosciencedbc.jp/en/) under research ID hum0014, Japanese Genotype-phenotype Archive (JGA; https://www.ddbj.nig.ac.jp/jga/index.html) under accession ID JGAS00000000114. Allele frequency data of this WGS data is publicly available at Japanese ENcyclopedia of GEnetic associations by Riken (JENGER; http://jenger.riken.jp/). WGS data of the rest of the BBJ subjects (*n* = 913) is available on request after approval of the ethical committee of RIKEN Yokohama Institute and the Institute of Medical Science. WGS data of the controls (*n* = 295) will be available on request under the condition of approval of the ethical committee of Keio University and material transfer agreement.

## Electronic supplementary material

Supplementary Information
