# Supplementary material for: Deep whole-genome sequencing reveals recent selection signatures linked to evolution and disease risk of Japanese
Source: Nat Commun. 2018 Apr 24;9:1631. doi: 10.1038/s41467-018-03274-0 (PMC5915442; doi:10.1038/s41467-018-03274-0)
Supplement: Supplementary file 1 — Supplementary Information [file 41467_2018_3274_MOESM1_ESM.pdf]

Deep whole-genome sequencing reveals recent selection signature linked to evolution and disease risk of Japanese

Okada Y et al.

Supplementary Table 1. Characteristics of the whole-genome sequence datasets

Datasets	No. subjects	Subject information	Institutes where WGS were conducted	WGS platform	DNA Library Preparation	Read length	Depth	No. variants	Ti/Tv ratio
Dataset 1	1,276	Participants of BBJ (acute myocardial infarction, drug eruption, colorectal cancer, breast cancer, prostate cancer, gastric cancer)	RIKEN Center for Integrative Medical Sciences	HiSeq 2500	TruSeq Nano DNA Library Preparation Kit	2×160 and 2×125-bp paired end (*n* = 1,026 and 250, respectively)	24.30	30,422,188	2.137
Dataset 2	492	Participants of BBJ (dementia, *n* = 197)	Toshiba Corporation	HiSeq X Five	TruSeq DNA PCR-Free Library Preparation Kit	2×150-bp paired end	36.57	20,830,514	2.114
		Japanese controls who lived over 100 years (*n* = 295)	Takara Bio Inc.	HiSeq 2500	TruSeq DNA PCR-Free Library Preparation Kit	2×125-bp paired end			
Dataset 3	466	Participants of from BBJ (acute myocardial infarction)	Macrogen Japan Corporation	HiSeq X Five	TruSeq DNA PCR-Free Library Preparation Kit	2×150-bp paired end	19.18	19,228,253	2.140
Total	2,234	-		-		-	25.93	39,898,568	-

WGS; whole-genome sequence, BBJ; the BioBank Japan Project.

Supplementary Table 2. SNPs with very recent selection signatures in the Japanese population

rsID				rs75721934	rs58008302	rs3782886	rs4822159
Chr				4	6	12	22
Position (hg19)				100,142,780	29,493,261	112,110,489	42,932,013
Ancestral/derived allele				G/A	G/A	T/C	C/G
Derived allele frequency (DAF) in WGS				0.750	0.186	0.289	0.193
Gene				ADH clusters	MHC region	*BRAP-ALDH2*	*SERHL2*
Selection signature z-score (SDS)				7.13	8.14	8.13	-5.80
Selection signature *P* (SDS)				9.7×10^{-13}	4.1×10^{-16}	4.4×10^{-16}	6.6×10^{-9}
DAF	1000 Genomes Project (phase3v5)	1KG global (*n* = 2,504)		0.174	0.095	0.046	0.243
		1KG AFR (*n* = 661)		0.001	0.123	0.000	0.424
		1KG AMR (*n* = 347)		0.000	0.042	0.003	0.120
		1KG EAS (*n* = 504)		0.672	0.129	0.175	0.156
		1KG EUR (*n* = 503)		0.000	0.012	0.000	0.187
		1KG SAS (*n* = 489)		0.001	0.063	0.000	0.211
		1KG EAS-CDX (*n* = 93)		0.601	0.070	0.043	0.081
		1KG EAS-CHB (*n* = 103)		0.670	0.107	0.155	0.141
		1KG EAS-CHS (*n* = 105)		0.714	0.057	0.271	0.152
		1KG EAS-JPT (*n* = 104)		0.731	0.216	0.250	0.245
		1KG EAS-KHV (*n* = 99)		0.626	0.192	0.136	0.152
	BioBank Japan Project	All regions (*n* = 171,176)		0.747	0.182	0.260	0.178
		Hokkaido (*n* = 7,910)		0.737	0.172	0.228	0.176
		Tohoku (*n* = 10,907)		0.743	0.150	0.203	0.183
		Kanto-Koshinetsu (*n* = 95,272)		0.755	0.196	0.271	0.174
		Chubu-Hokuriku (*n* = 9,390)		0.761	0.206	0.293	0.181
		Kinki (*n* = 26,160)		0.757	0.190	0.289	0.164
		Kyushu (*n* = 15,818)		0.726	0.154	0.229	0.192
		Okinawa (*n* = 5,719)		0.646	0.037	0.137	0.263
DAF heterogeneity		1000 Genomes Project global	Fold change	10.26	0.61	3.66	0.99
			P	8.5×10^{-6}	0.63	0.0044	0.40
		BioBank Japan Project	Fold change	4.32	14.82	12.17	3.41
			P	0.021	7.4×10^{-5}	1.5×10^{-5}	0.045

AFR; African, AMR; Admixed American, EAS; East Asian, EUR; European, SAS; South Asian, CDX; Chinese Dai in Xishuangbanna, CHB; Han Chinese in Beijing, CHS; Southern Han Chinese, JPT; Japanese in Tokyo, KHV; Kinh in Ho Chi Minh City.

Supplementary Table 3. Enrichment of SDS selection signatures on Japanese GWAS-associated SNPs

Traits in Japanese genome-wide association studies (GWAS)	Trait category	No. GWAS risk variants	SDS signal enrichment		
			χ^2 value inflation	*P*	
Drinking	Behavior	2	20.46	1.3E-09	* †
Esophageal cancer	Disease	2	20.46	1.3E-09	* †
Serum chloride	Electrolyte	17	3.89	9.9E-08	* †
Serumalpha1-antitrypsinlevels	Other biochemical	3	9.76	2.0E-06	* †
Total protein	Protein	32	2.54	3.7E-06	* †
Alanine aminotransferase	Liver-related	26	2.71	5.7E-06	* †
Gout	Disease	5	6.31	7.3E-06	* †
Red blood cell count	Hematological	46	2.17	7.7E-06	* †
Blood sugar	Metabolic	16	3.16	1.8E-05	* †
Acute myocardial infarction	Disease	3	7.98	2.6E-05	* †
Aspartate aminotransferase	Liver-related	24	2.61	2.7E-05	* †
Low density lipoprotein cholesterol	Metabolic	21	2.74	2.9E-05	* †
Uric acid	Kidney-related	26	2.43	6.2E-05	* †
Activated partial thromboplastin time	Other biochemical	9	3.78	9.0E-05	* †
Serum sodium	Electrolyte	13	3.15	9.9E-05	* †
High density lipoprotein cholesterol	Metabolic	29	2.28	0.00010	* †
Narcolepsy	Disease	2	9.08	0.00011	* †
Height	Anthropometric	254	1.35	0.00018	* †
CD8+ T cell eQTL	Immune cell eQTL	1,599	1.13	0.00030	* †
Gamma-glutamyl transferase	Liver-related	42	1.87	0.00056	†
Takayasu's arteritis	Disease	2	7.45	0.00058	†
Natural killer cell eQTL	Immune cell eQTL	1,517	1.12	0.00092	†
Estimated glomerular filtration rate	Kidney-related	65	1.62	0.0011	†
Ulcerative colitis	Disease	5	3.91	0.0015	†
Albumin/globulin ratio	Protein	38	1.82	0.0015	†
Diastolic blood pressure	Blood pressure	16	2.36	0.0016	†
Body mass index	Anthropometric	63	1.60	0.0018	†
Non-albumin protein	Protein	44	1.73	0.0018	†
Systolic blood pressure	Blood pressure	22	2.09	0.0020	†
Total cholesterol	Metabolic	32	1.85	0.0025	†
Triglyceride	Metabolic	24	2.00	0.0025	†
CD4+ T cell eQTL	Immune cell eQTL	1,826	1.09	0.0039	†
Neutrophil count	Hematological	21	2.01	0.0040	†
Mean corpuscular hemoglobin concentration	Hematological	34	1.76	0.0041	†
Pulse pressure	Blood pressure	17	2.14	0.0042	†
Albumin	Protein	17	2.12	0.0046	†
Platelet count	Hematological	72	1.48	0.0053	†
Mean arterial pressure	Blood pressure	27	1.78	0.0075	†
Adult asthma	Disease	5	3.09	0.0087	†
EGFR-positive lung cancer	Disease	7	2.69	0.0088	†
Peripheral blood eQTL	Immune cell eQTL	1,918	1.08	0.011	†
Mean corpuscular hemoglobin	Hematological	74	1.40	0.014	†
B cell eQTL	Immune cell eQTL	1,669	1.08	0.014	†
Mean corpuscular volume	Hematological	82	1.36	0.016	†
Serum creatinine	Kidney-related	63	1.42	0.016	†
Lactate dehydrogenase	Other biochemical	10	2.07	0.023	
Hemoglobin	Hematological	16	1.74	0.032	
Serum potassium	Electrolyte	12	1.86	0.034	
White blood cell count	Hematological	34	1.48	0.036	
Hemoglobin A1c	Metabolic	23	1.57	0.040	
Atopic dermatitis	Disease	8	2.01	0.041	
Monocyte eQTL	Immune cell eQTL	2,166	1.05	0.066	
Systemic lupus erythematosus	Disease	6	1.87	0.082	
Basophil eQTL	Hematological	25	1.41	0.083	
Alkaline phosphatase	Liver-related	25	1.39	0.093	
Breast cancer	Disease	1	2.32	0.13	
Rheumatoid arthritis	Disease	44	1.23	0.14	
Kawasaki disease	Disease	4	1.73	0.14	
Serum phosphorus	Electrolyte	7	1.54	0.15	
Lymphocyte count	Hematological	12	1.40	0.16	
Crohn's disease	Disease	6	1.54	0.16	
Age-related macular degeneration	Disease	4	1.43	0.22	
Prothrombin time	Other biochemical	8	1.32	0.23	
C-reactive protein	Other biochemical	7	1.34	0.23	
Breast cancer chemotherapy adverse event	Disease	1	1.45	0.23	
Type 2 diabetes	Disease	31	1.17	0.23	
Total bilirubin	Liver-related	11	1.25	0.25	
Colon cancer	Disease	2	1.39	0.25	
Serum calcium	Electrolyte	14	1.19	0.27	
Left ventricular internal dimension in diastole	Echocardiographic	3	1.29	0.27	
Scoliosis	Disease	4	1.24	0.29	
Psoriasis vulgaris	Disease	2	1.20	0.30	
Blood urea nitrogen	Kidney-related	41	1.10	0.31	
Moyamoya disease	Disease	1	0.99	0.32	
Nonalcoholic fatty liver disease	Disease	1	0.95	0.33	
Primary biliary cirrhosis	Disease	6	1.05	0.39	
Hematocrit	Hematological	19	1.04	0.41	
Relative wall thickness	Echocardiographic	1	0.63	0.43	
Parkinson's disease	Disease	4	0.91	0.45	
Bladder cancer	Disease	1	0.56	0.45	
Peripheral artery disease	Disease	3	0.85	0.47	
Fibrinogen	Other biochemical	1	0.53	0.47	
Behcet 's disease	Disease	3	0.84	0.47	
Prostate-specificantigenlevels	Other biochemical	2	0.73	0.48	
Uterine fibroids	Disease	2	0.72	0.49	
Eosinophil eQTL	Hematological	18	0.96	0.50	
Smoking	Behavior	1	0.45	0.50	
Ejection fraction	Echocardiographic	2	0.67	0.51	
Creatine kinase	Other biochemical	35	0.93	0.59	
Left ventricular internal dimension in systole	Echocardiographic	3	0.57	0.63	
Fractional shortening	Echocardiographic	3	0.56	0.64	
Zinc sulfate turbidity test	Liver-related	4	0.62	0.65	
Keloid	Disease	3	0.48	0.70	
Stroke	Disease	5	0.57	0.72	
Prostate cancer	Disease	14	0.66	0.82	
Alzheimer's disease	Disease	2	0.18	0.84	
Exfoliation syndrome	Disease	2	0.16	0.85	
Warfarin maintenance dose	Pharmacogenetics	3	0.17	0.92	
Stevens-Johnson syndrome by cold medicine	Disease	5	0.28	0.93	
Response to hepatitis C treatment	Pharmacogenetics	3	0.07	0.98	
Ossification of the posterior longitudinal ligament	Disease	6	0.18	0.98	
Atrial fibrillation	Disease	16	0.31	1.00	

*: Bonferroni's correction *P* < 0.05, †: false-discovery rate (FDR)-*q* < 0.05.

Supplementary Figure 1. Alternative allele frequency spectra of the whole-genome sequence (WGS) data of the Japanese population

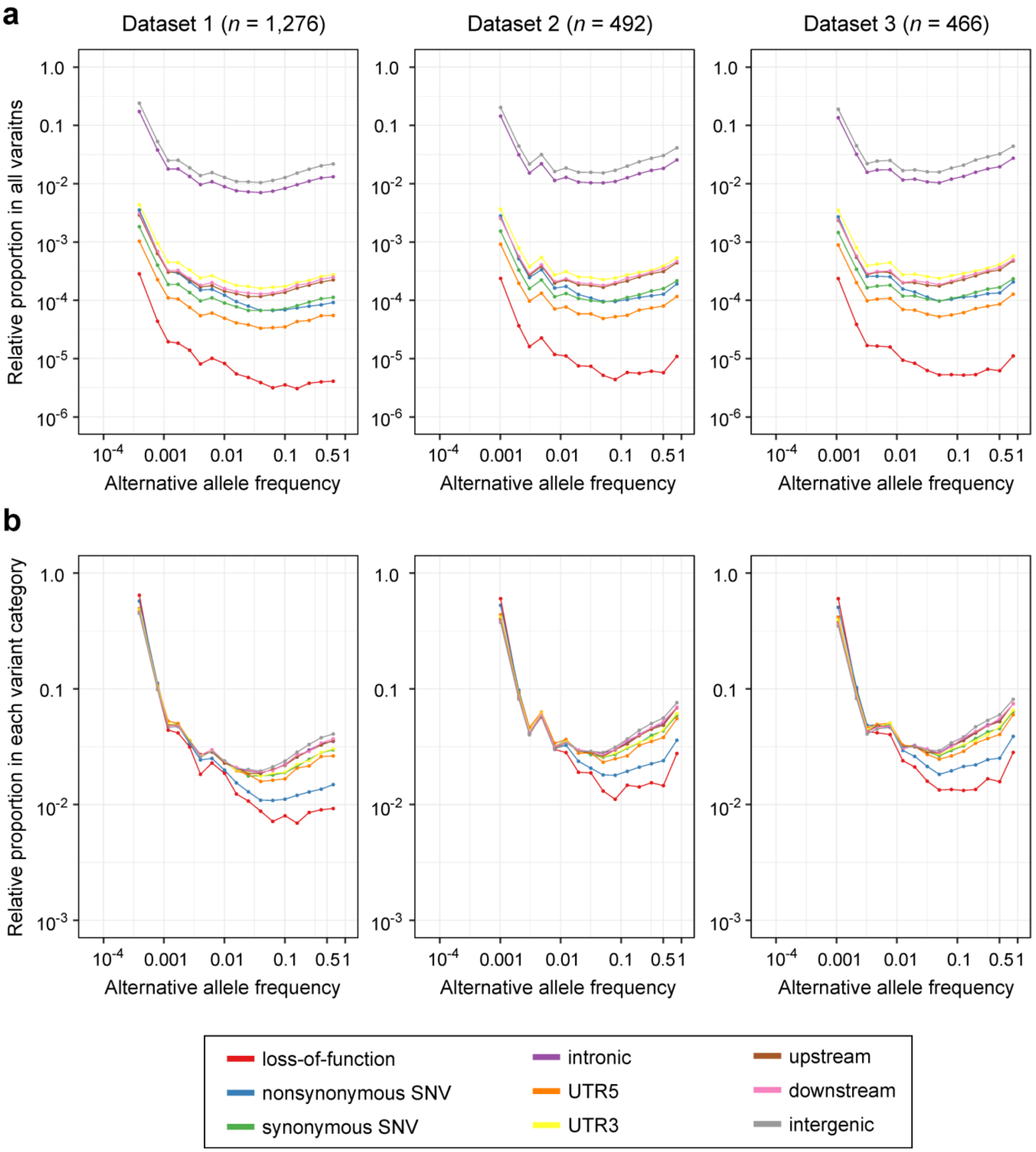

Alternative allele frequency spectra of the variants identified in the whole genome sequence (WGS) analysis of the Japanese population. Relative proportions (**a**) in all variants and (**b**) in each variant category based on functional annotation by ANNOVAR are indicated separately for the datasets 1-3.

Supplementary Figure 2. Alternative allele frequency spectra of the whole-genome sequence (WGS) data of the worldwide populations

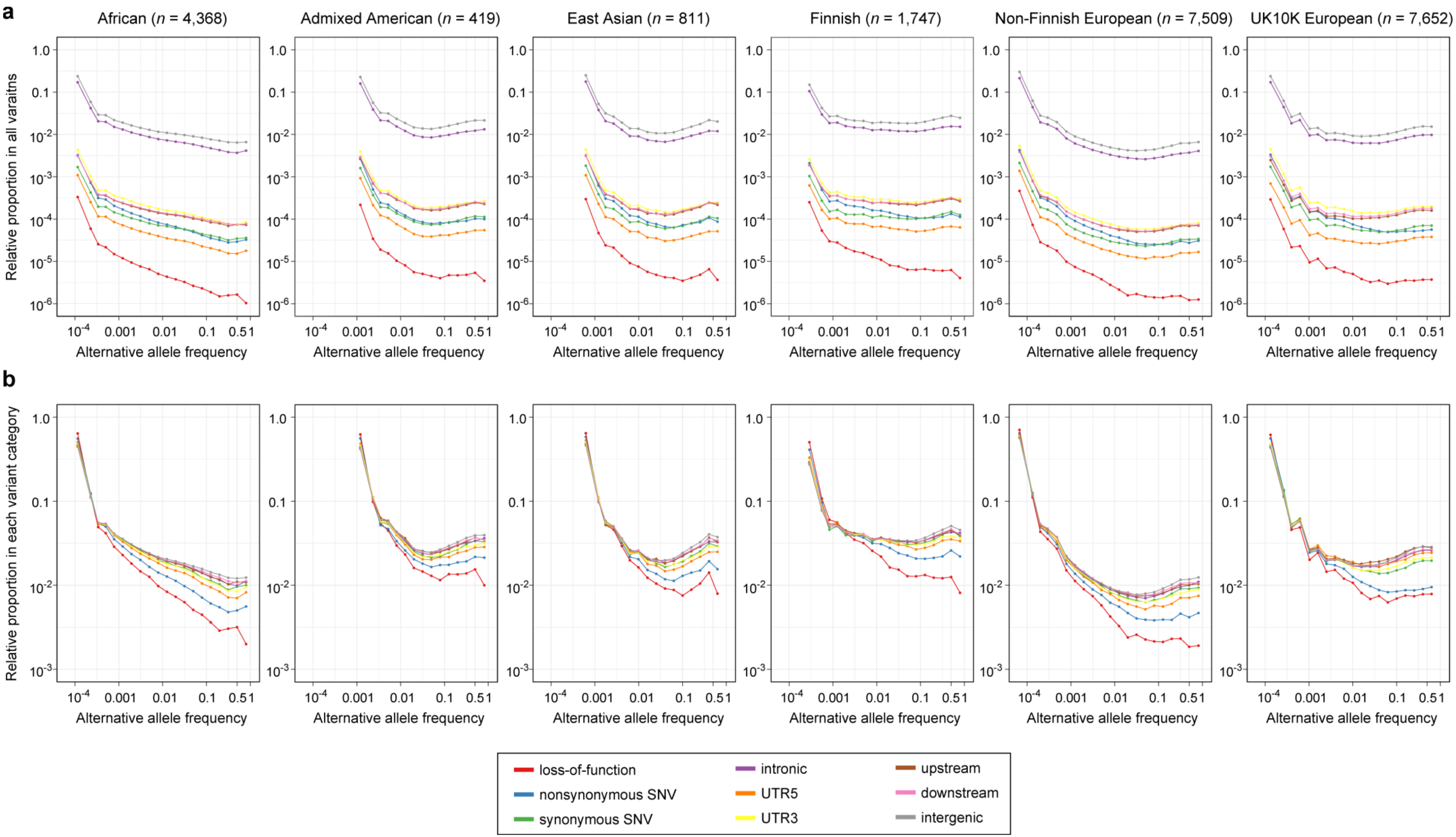

Alternative allele frequency spectra of the variants identified in the whole genome sequence (WGS) analysis of the worldwide populations. Relative proportions (**a**) in all variants and (**b**) in each variant category based on functional annotation by ANNOVAR are indicated separately.

Supplementary Figure 3. A quantile-quantile (QQ) plot of the genome-wide natural selection signatures in Japanese

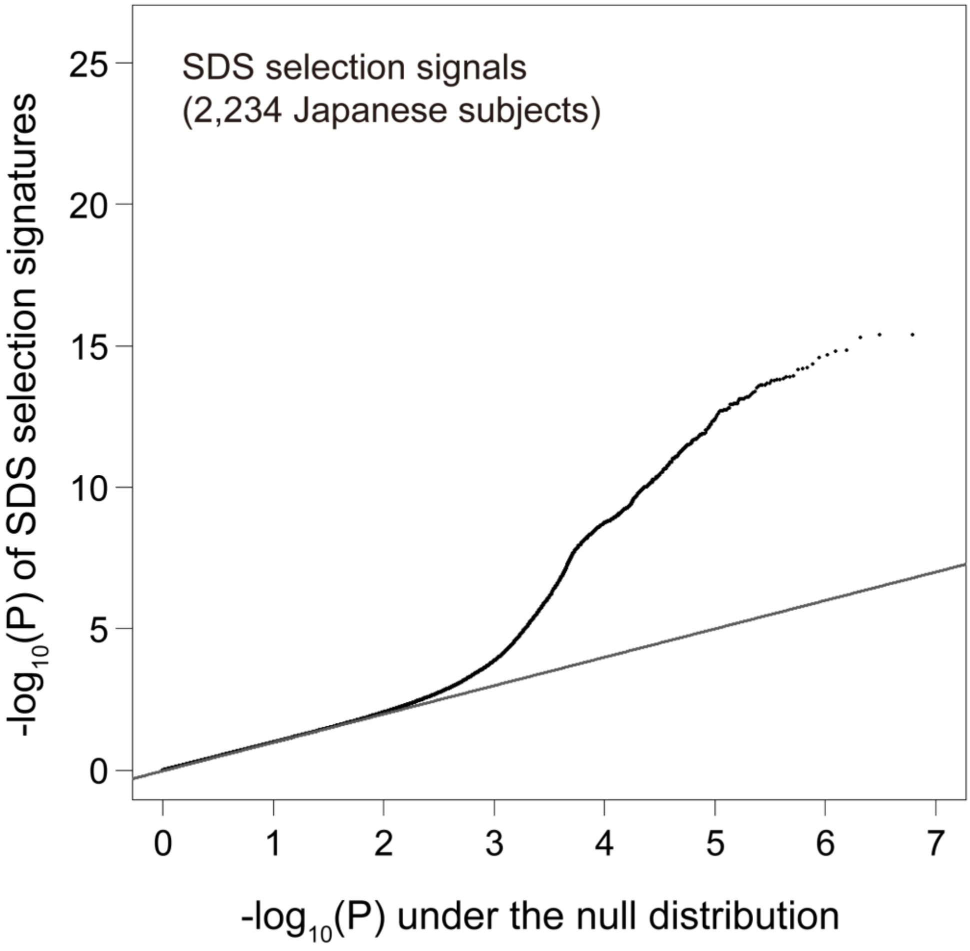

A quantile-quantile (QQ) plot of the genome-wide natural selection signatures obtained from the WGS data of the 2,234 Japanese individuals. The *x*-axis and *y*-axis indicate the expected and observed distributions of the $-\log_{10}(P)$ of genome-wide selection signatures calculated by using SDS.

Supplementary Figure 4. Linkage disequilibrium (LD) blocks and regional plots of the genetic loci with significant natural selection signatures in Japanese

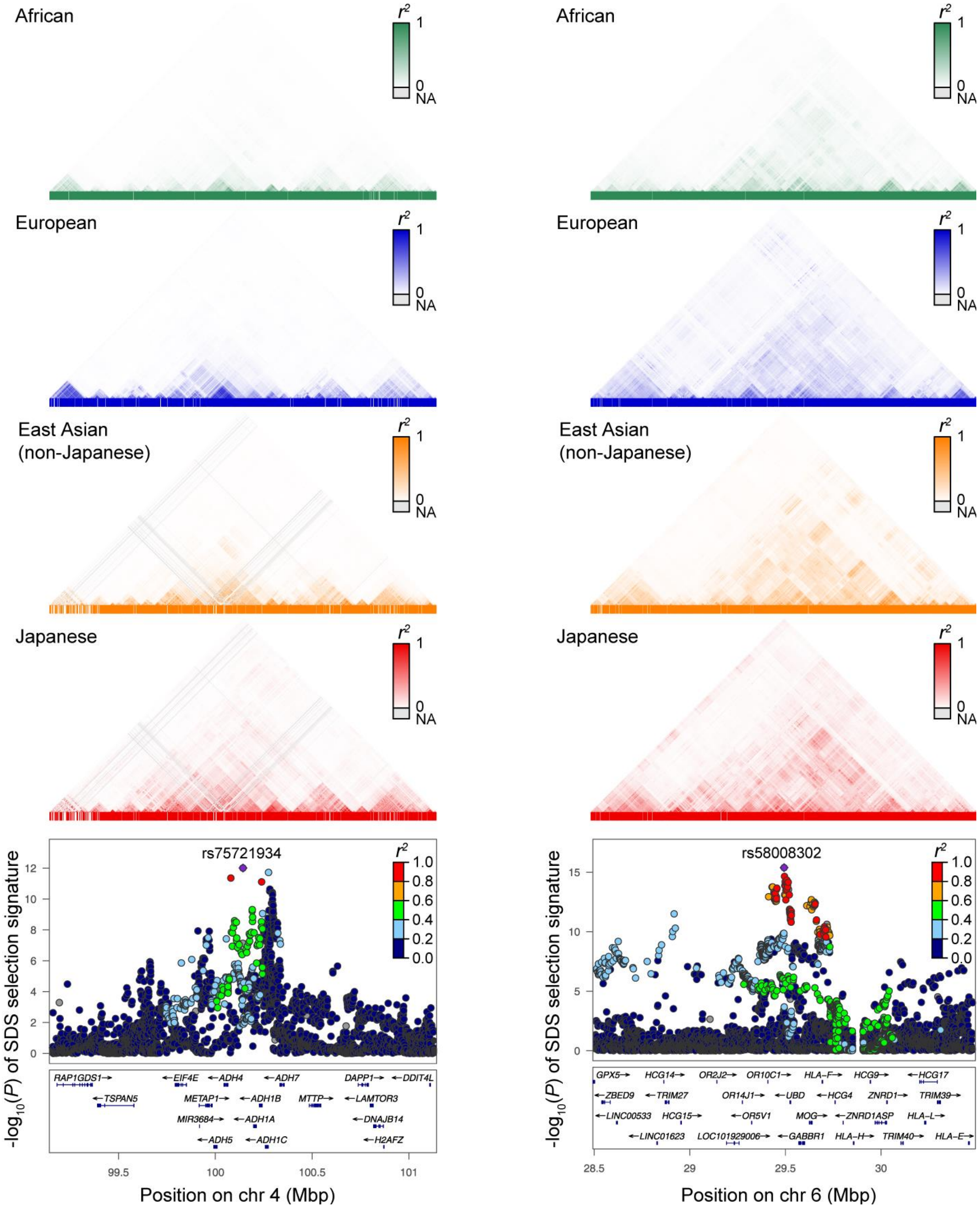

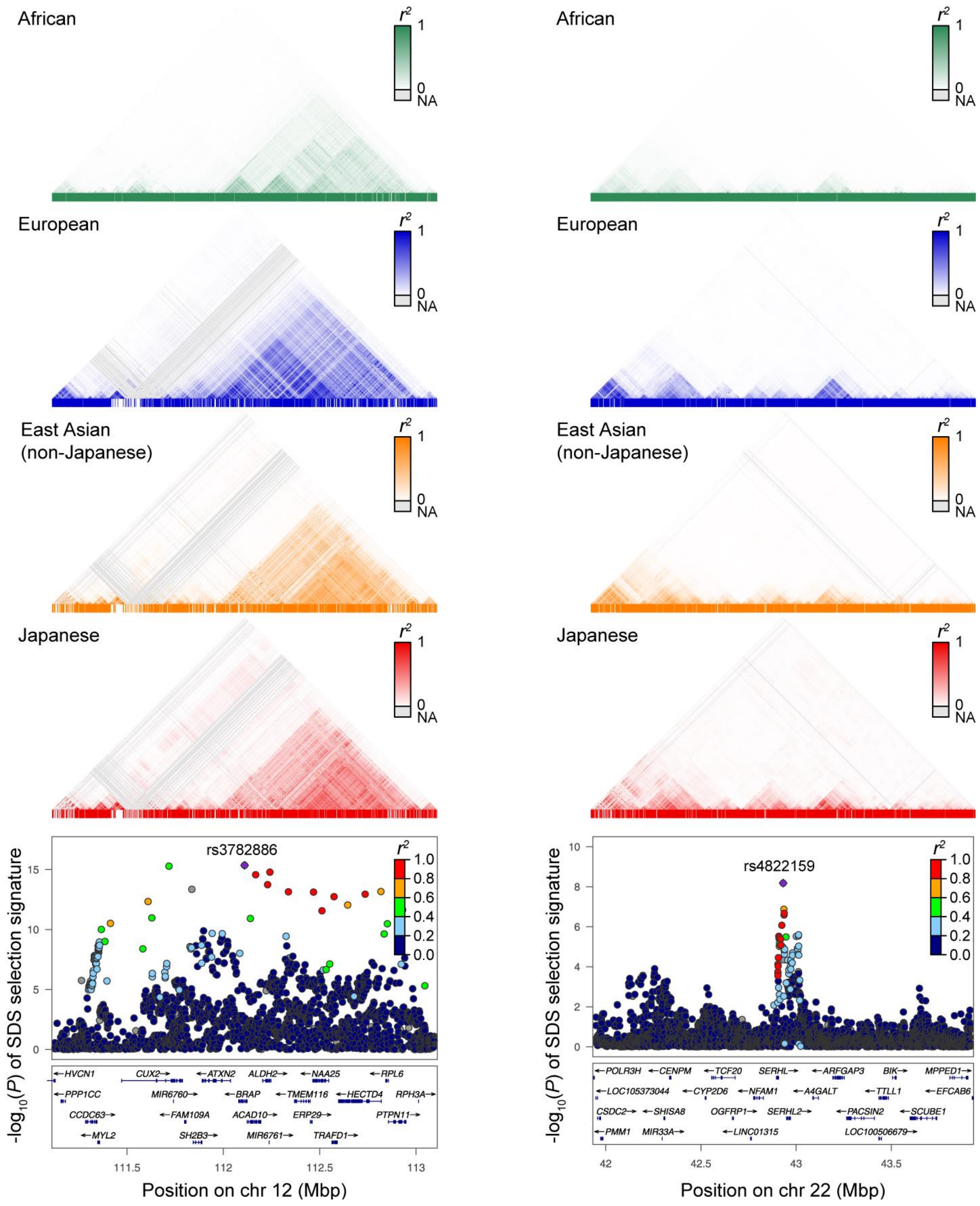
African
European
East Asian (non-Japanese)
Japanese
r^2
1
0
NA
rs3782886
rs4822159
1.0
0.8
0.6
0.4
0.2
0.0
-log$_{10}$(P) of SDS selection signature
HVCN1
CUX2
ATXN2
ALDH2
NAA25
RPL6
PPP1CC
MIR6760
BRAP
TMEM116
HECTD4
RPH3A
CCDC63
FAM109A
ACAD10
ERP29
PTPN11
MYL2
SH2B3
MIR6761
TRAFD1
111.5
112
112.5
113
Position on chr 12 (Mbp)
POLR3H
CENPM
TCF20
SERHL
ARFGAP3
BIK
MPPED1
LOC105373044
CYP2D6
NFAM1
A4GALT
TTLL1
EFCAB6
CSDC2
SHISA8
OGFRP1
SERHL2
PACSIN2
SCUBE1
PMM1
MIR33A
LINC01315
LOC100506679
42
42.5
43
43.5
Position on chr 22 (Mbp)

Each panel indicates the linkage disequilibrium (LD) blocks (the upper four panels) and the regional plots (the lowest panel) of the genetic loci with significant natural selection signatures in Japanese. LD blocks were drawn based on the r^2 values calculated from the subjects in the 1000 Genomes Project phase 3v5 database. Regional plots of the natural selection signature p-values of SDS were obtained from LocusZoom.

Supplementary Figure 5. Principal component plots of the Japanese population in each of the geographic regions

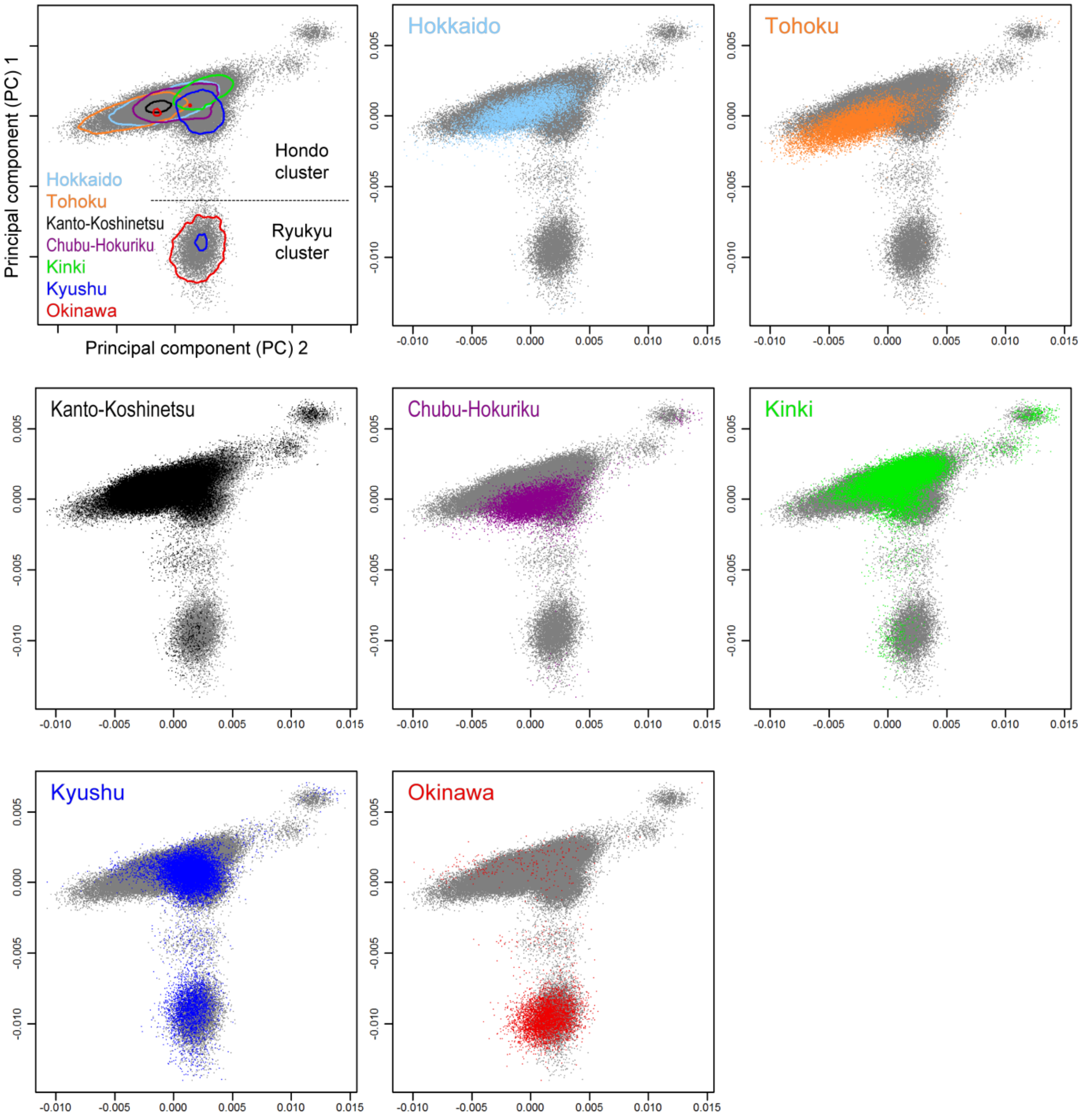

Individuals of each of the Japanese regions are separately colored on the principal component (PC) plots consisting of PC1 and PC2.

Supplementary Figure 6. Enrichment of selection signatures in archaic hominins-derived genome sequences

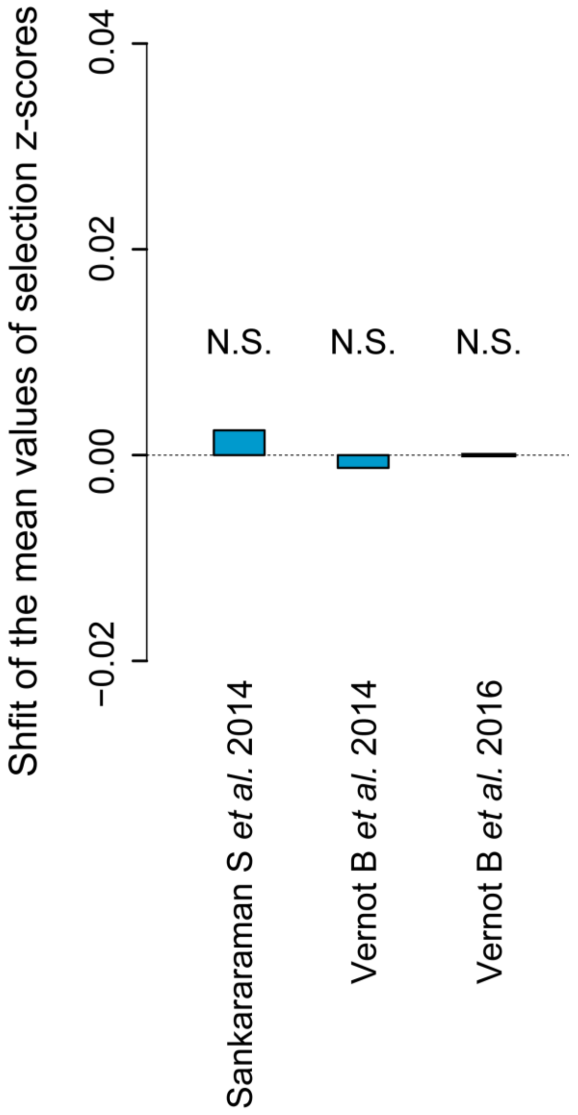

Shift of the mean values of the normalized SDS z-scores within genome sequences derived from archaic hominins are indicated along the *y*-axis. We evaluated three sets of Neanderthal-derived sequences (Sankararaman S *et al.* 2014, Vernot B *et al.* 2014, Vernot B *et al.* 2016). No significant shift of the selection z-score mean values from zero was observed (permutation $P > 0.30$). We note that no Denisovan-derived sequences observed in the Japanese population, and thus, enrichment analysis was not done.

Supplementary Figure 7. Overlap between natural selection signatures and genetic risk of human phenotypes in Japanese (after removal of the region with genome-wide significant selection signature)

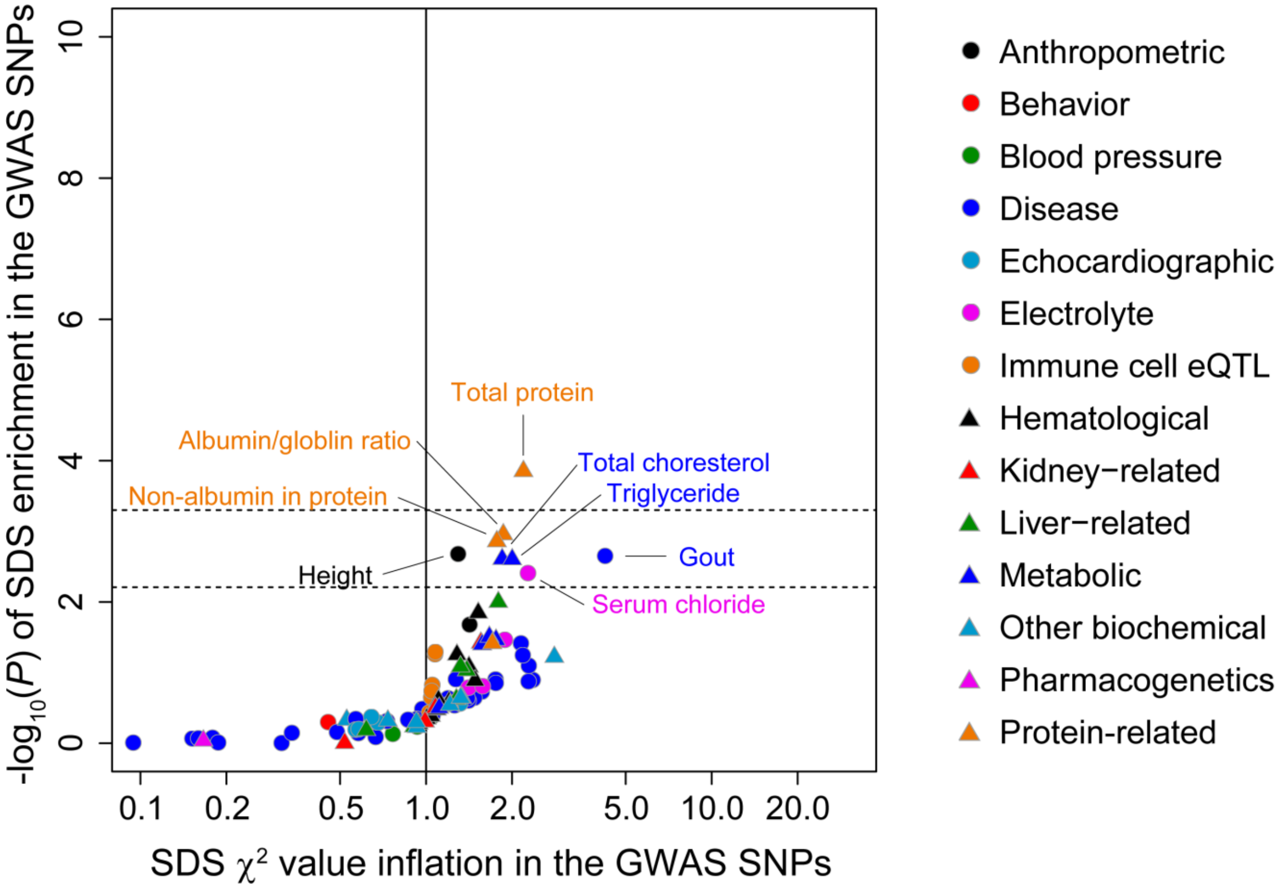

Enrichment of the natural selection signatures in the GWAS-associated variants of the diseases (n = 36), quantitative traits (n = 61), and immune-cell specific eQTL (n = 6) in Japanese. Enrichment was assessed after removal of the genomic loci that indicated genome-wide significant selection signature in the SDS analysis (i.e., ADH clusters, MHC region, *BRAP1-ADLH2*, and *SERHL2*). For each trait, inflation of the selection χ^2 value is indicated along the x-axis, and $-\log_{10}(P)$ of enrichment is plotted along the y-axis. The horizontal gray lines represent significance threshold based of Bonferroni's correction on the numbers of the evaluated traits (P < 0.00049, upper) and false-discovery rate (FDR)-q < 0.05 (lower). The traits which satisfied FDR-q < 0.05 are labeled.
